# Microbiome Landscapes in Squamous Cell Carcinoma Tissue Microenvironments: A Comparative Analysis

**DOI:** 10.1002/iid3.70406

**Published:** 2026-03-17

**Authors:** Ruiqian Yao, Fang Qian, Haixia Zhao, Xiaomin Shen, Yuanjie Zhu, Liangzhe Wang

**Affiliations:** ^1^ Department of Dermatology, Naval Medical Centre Naval Medical University Shanghai China; ^2^ Tongren Hospital Shanghai Jiao Tong University School of Medicine Shanghai China

**Keywords:** Cancer microbiome, RNA sequencing, squamous cell carcinoma, tumor‐associated microbiota

## Abstract

**Introduction:**

Squamous cell carcinoma (SCC) is one of the most prevalent human cancers. While anatomically distinct, SCCs exhibit diverse similarities in etiology and molecular. The extent to which different SCCs share microbial landscapes within the tumor tissue microenvironment remains unclear.

**Methods:**

We analyzed RNA sequencing data from 419 SCC samples across five anatomical sites: cutaneous (CuSCC), esophageal (ESCC), lung (LSCC), head and neck (HNSCC), and cervical (CeSCC). Differential microbial abundance between tumor and adjacent normal tissue was assessed using multivariable linear models implemented in MaAsLin2. For each anatomical site, an independent external validation cohort was included (totaling 156 samples) to validate key microbial findings using the area under the receiver operating characteristic curve (AUROC).

**Results:**

The five SCC cohorts shared 28 shared core bacterial genera, with *Staphylococcus* was widespread and had the highest relative abundance (mean 13.74%) in all cohorts. ESCC, HNSCC, and LSCC exhibited more similar dysregulated microbiota, with *Clostridioides* showing the most significant up‐regulation in tumor relative to adjacent tissue (the mean model coefficient value, coef = 3.26). Notably, *Bradyrhizobium* (CeSCC, CuSCC, LSCC, mean coef = −2.58), *Massilia* (in CeSCC, ESCC, HNSCC, mean coef = −1.99), *Providencia* (in CeSCC, HNSCC, LSCC, mean coef = 0.93), and *Ralstonia* (in CuSCC, ESCC, HNSCC, mean coef = −0.52) displayed significant differential expression across multiple cohorts, as confirmed in the validation cohorts (AUROC > 0.6).

**Conclusions:**

Despite the absence of a common dysbiotic microbiota among SCCs due to anatomical differences, potential similarities in adjacent sites suggest a unified disease perspective and may pave the way for novel preventive and therapeutic strategies.

## 1 Introduction

The human body is home to an estimated three trillion bacterial members that coordinate a comprehensive interplay of physiological processes and disease susceptibility [1]. There is a growing interest in the important role of microorganisms in maintaining health and in the onset of diseases. The involvement of specific microorganisms, including bacteria as well as viruses, in the pathogenesis of cancer has been the subject of extensive research [2]. For instance, the notorious *Helicobacter pylori* has been shown to induce degradation of the p53 protein in gastric epithelial cells, thereby promoting gastric cancer [3]. Similarly, *Fusobacterium nucleatum* has been found to inhibit the tumoricidal activity of natural killer (NK) cells against various malignancies [4]. While most research has concentrated on the gut microbiome and its relationship with cancer progression and treatment, emerging studies have demonstrated that in solid tumors, the presence of microorganisms in the tumor and adjacent normal tissues can also provide insights into disease progression and their role in cancer pathogenesis [5].

Squamous cell carcinoma (SCC), one of the most common human solid tumors, originates from squamous epithelial cells that form the surface layer of the skin and line many internal organs, including the esophagus, lung, head and neck, and cervix. Despite their classification by anatomical origin, SCCs exhibit shared genomic, genetic, and epigenetic features, suggesting a unified biological framework that could inform the development of common and innovative therapeutic strategies for these widespread and challenging cancers [6].

Next‐generation sequencing (NGS) technologies, such as RNA sequencing (RNA‐seq), have deepened our understanding of the human microbiome by uncovering and characterizing previously unculturable microbes and predicting their function [7]. Currently, there is a huge amount of raw sequencing data archived in public databases, such as the National Center for Biotechnology Information (NCBI) Sequence Read Archive (SRA) database, waiting to be mined. In this study, we retrieved raw RNA‐seq data of SCC from five distinct anatomical sites, including the cervix, esophagus, head and neck, lung, and skin, from the NCBI SRA database, aiming to explore the similarities and differences of the microbiomes within the tumor microenvironment of SCC. The insights gleaned from this study might potentially pave the way for novel diagnostic markers and microbiome‐targeted therapeutic approaches, ultimately enhancing the prognosis of SCC patients.

## 2 Methods

### 2.1 Data Collection

We collected SCC RNA‐seq data from the NCBI SRA database for five anatomical sites including cervix, esophagus, head and neck, lung, and skin, namely five cohorts of cutaneous SCC (CuSCC), esophageal SCC (ESCC), lung SCC (LSCC), head and neck SCC (HNSCC), and cervical SCC (CeSCC). The SRA database was searched using the keywords, “cutaneous/skin squamous cell carcinoma”, “esophageal squamous cell carcinoma”, “lung squamous cell carcinoma”, “head and neck squamous cell carcinoma”, and “cervical squamous cell carcinoma”. For each cohort, we ensured that two datasets were available for analysis and one dataset for validation. Each dataset included samples from both tumor and adjacent normal tissues. Gender information was not available for all samples as this metadata was not reported in the original SRA submissions.

### 2.2 Identification and Quantification of the Microbiome

The raw data from all projects were downloaded in the SRA format and converted into fastq format using the SRA Toolkit (version 3.0.1). The raw fastq files were processed for quality control using the software FastQC (version 0.11.9) and Trim Galore (version 0.6.7). Reads were trimmed to remove adapters and low‐quality bases with Phred score less than 20, and reads shorter than 20 bp after trimming were discarded. The ultrafast Karen2 algorithm was used to identify the microbes [8]. The microbial reference database contains 83,212 genomes, which include almost all known fungal, bacterial, archaeal, and viral genomes. Kraken2 processes the paired‐end fastq files using default parameters (with the confidence score threshold set to 0.0). Finally, the assigned taxa were aggregated at the genus level and the relative abundance was calculated, which is the fraction of the taxon observed in the feature table relative to the sum of all taxa in the sample (ranging from 0 to 1) [9], for follow‐up analysis.

### 2.3 Batch Effect Removal

This study combined the two datasets used for analysis for each cohort to reduce the impact of individual differences on the results. Conditional Quantile Regression (ConQuR), a method developed specifically for microbiome data, removed the batch effect [10]. ConQuR removes batch effects on a taxa‐by‐taxa and sample‐by‐sample basis using a two‐part quantile regression model to account for the zero‐inflated and over‐dispersed distribution of microbial read counts. Specifically, a logistic regression component models the presence or absence of each taxon, while conditional quantile regression models the distribution of read counts given taxon presence. Batch effects are estimated and removed relative to a reference batch while simultaneously adjusting for relevant covariates. During batch correction, biologically meaningful signals related to tissue source (tumor and adjacent normal tissue), were retained to ensure that downstream analyses reflect true biological differences rather than technical variation. This framework allows batch effects to vary across different abundance levels rather than assuming a uniform shift, thereby preserving complex distributional features of microbiome data.

PCoA (principal coordinates analysis) analysis shows the heterogeneity between different data sets before and after correction.

### 2.4 Microbial Profile Analysis

Alpha diversity (Shannon index) and Bray‐Curtis distance were calculated using vegan R‐package (version 2.6–2). The phyloseq (version 1.48.0) and microbiome (version 1.26.0) R‐packages were used to summarise taxa to phylum and genus level and to calculate relative abundance. Differential analysis was determined in diversity using the Wilcoxon signed rank test. PERMANOVAR was used to quantify multivariate community‐level differences in microbial composition among groups. *p*‐value < 0.05 was considered significant at the group level.

Core microbiota were identified using the microbiome R package (version 1.28.0). Core taxa were defined as those consistently present across samples at a minimum positive detection rate and prevalence threshold. Specifically, the positivity detection rate was set to 0.1%, and taxa were required to occur in at least 10% of samples. Visualization of shared taxa was performed with the Venn (version 1.12) and UpSet (version 1.4.0) R‐packages.

### 2.5 Differential Abundance Analysis

For the analysis cohort, microbial abundance data were normalized using the trimmed mean of M‐values (TMM) method to correct for differences in library size across samples. Statistically significant differences among taxa were performed using Microbiome Multivariable Association with Linear Models (MaAsLin2), a multivariable linear modeling framework that accommodates sparse, compositional microbiome data, adjusts for covariates and repeated measures, and controls false discovery rates [11]. Only taxa with *p*‐value < 0.05 were considered significantly enriched.

### 2.6 Validation Cohort Analysis

In the validation cohort, microbial abundance data were also normalized using the trimmed mean of M‐values (TMM) method. The pROC R‐package (version 1.18.2) was used to plot the receiver operating characteristic (ROC) curve and the area under the curve (AUC) was calculated to evaluate the ability of taxa that showed significant differences in the analysis cohort to discriminate between tumor and adjacent normal tissue samples. The Area Under the Receiver Operating Characteristic Curve (AUROC) > 0.6 is considered to have the ability to distinguish tumor tissue from adjacent tissue.

## 3 Result

### 3.1 Cohort Characteristics

We collected datasets of five cohorts, including CuSCC, ESCC, LSCC, HNSCC, and CeSCC, from the NCBI SRA database, and the cohort information is shown in Table 1. A total of 575 samples were used in this study, of which 72.87% (419 samples) were used for analysis and the remaining samples (156 samples) were used for validation. The number of tumor and adjacent normal tissue samples was 54.43% and 45.57% of the total number of samples, respectively, and the sex ratio, based on samples with available gender data, was 5.69/1 (male/female). To reduce the impact of heterogeneity in the individual datasets on the results, we integrated the two datasets used for analysis in each cohort and PCoA showed that the ConQuR method significantly eliminated batch effects in the integrated data (Figure S1A–E).

**Table 1:** Cohort characteristics.

Site	Dataset	Project ID	Country	Proportion of the total samples	Gender			Tissue	
					Male	Female	Unknown	Tumor	Adjacent
CuSCC	Train	PRJNA437465	USA	8.11% (34/419)	/	/	18	8	10
		PRJNA790997	USA		/	/	16	8	8
	Validation	PRJNA515744	USA	12.18% (19/156)	/	/	19	10	9
ESCC	Train	PRJNA533799	South Korea	15.75% (66/419)	42	4	/	23	23
		PRJNA629358	USA		14	6	/	10	10
	Validation	PRJNA665149	China	23.08% (36/156)	30	6	/	18	18
LSCC	Train	PRJNA331133	Luxembourg	17.18% (72/419)	/	/	18	9	9
		PRJNA493790	China		33	2	19	35	19
	Validation	PRJNA670689	USA	18.59% (29/156)	/	/	29	17	12
HNSCC	Train	PRJNA524228	China	51.07% (214/419)	106	8	/	57	57
		PRJNA540754	China		94	6	/	50	50
	Validation	PRJNA739573	China	35.9% (56/156)	56	/	/	35	21
CeSCC	Train	PRJNA397117	China	7.88% (33/419)	/	6	/	3	3
		PRJNA793244	China		/	27	/	16	11
	Validation	PRJNA928409	USA	10.26% (16/156)	/	16	/	14	2
Total	Train	419 samples			289	59	71	219	200
	Validation	156 samples			86	22	48	94	62

### 3.2 Microbial Composition of SCC

**Figure 1:**
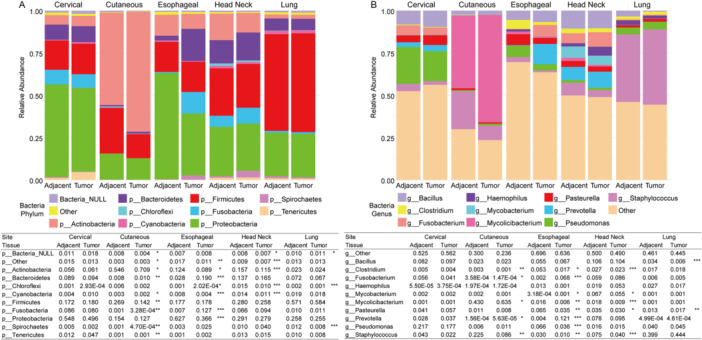
Stacked bar charts and tables showing the relative abundances of the top 10 phyla (A) and top 10 genera (B) in the five SCCs. Wilcoxon test, * indicates 0.01 < *p*‐value < 0.05, ** indicates 0.001 < *p*‐value < 0.01, *** indicates *p*‐value < 0.001, and blank indicates *p*‐value > 0.05.

On the phylum level, the phyla *Proteobacteria* (mean relative abundance 0.34), *Firmicutes* (mean relative abundance 0.28), and *Actinobacteria* (mean relative abundance 0.19) were the three predominant bacteria phyla found in SCC samples (Figure 1A). The genera *Staphylococcus* (mean relative abundance 0.14), *Mycolicibacterium* (mean relative abundance 0.11), and *Pseudomonas* (mean relative abundance 0.06) were the most abundant genera in SCC samples (Figure 1B).

### 3.3 Microbiota Dysbiosis in SCC

**Figure 2:**
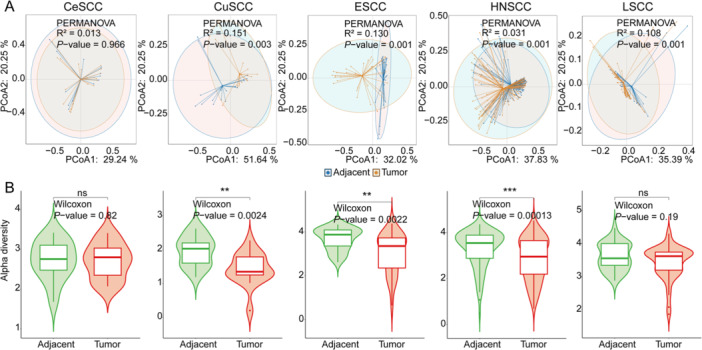
(A) POCA and (B) Alpha diversity analysis between tumor and adjacent normal tissue samples in CeSCC, CuSCC, ESCC, HNSCC, and LSCC cohorts.

From an overall perspective, PCoA revealed significant differences in microbial composition between tumor and adjacent tissues for CuSCC (R^2^ = 0.151, *p*‐value = 0.003), ESCC (R^2^ = 0.130, *p*‐value = 0.001), HNSCC (R^2^ = 0.031, *p*‐value = 0.001), and LSCC (R^2^ = 0.108, *p*‐value = 0.001). However, in CeSCC, the differences between tumor and adjacent tissues were not statistically significant (R^2^ = 0.013, *p*‐value = 0.966) (Figure 2A). Meanwhile, we calculated the Shannon index of bacteria for each SCC. It was found that the bacterial diversity in adjacent normal tissues was significantly higher than that in tumor tissues in CuSCC, ESCC and HNSCC, while it was not significant in CeSCC and LSCC (Figure 2B). The above results confirm that microbiota dysbiosis is present in the SCC tissue microenvironment.

### 3.4 Core Microbiota Characterization

**Figure 3:**
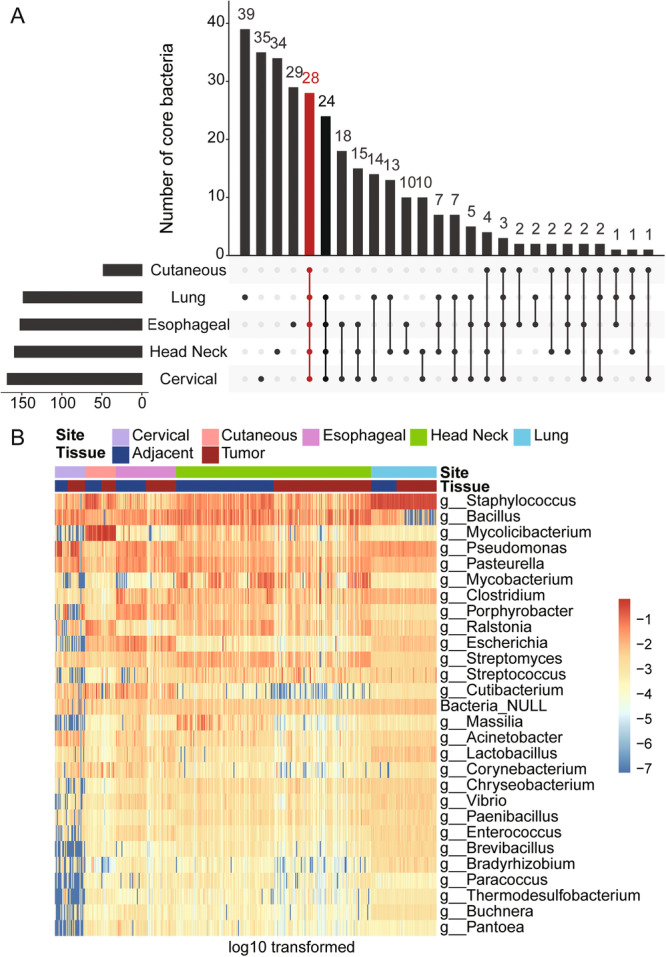
Frequency of shared core microbiota across five cohorts. (A) 28 bacteria (red highlight) were found to be shared across all five cohorts. (B) Heatmap of the relative abundance distribution of the shared core microbiota in the cohorts.

To identify differences and commonalities of genus shared between and across each cohort, the core microbiota was characterized. 48 (CuSCC), 152 (ESCC), 148 (LSCC), 159 (HNSCC), and 168 (CeSCC) genera were identified as core microbiota in each cohort, respectively. Among them, 28 shared bacterial genera represent 9.032% of the total microbiome diversity observed across all samples (Figure 3A). *Staphylococcus*, a Gram‐positive pathogenic bacterium, was the common core bacteria with the highest average relative abundance. This was followed by *Bacillus*, *Mycolicibacterium*, *Pseudomonas*, and *Pasteurella*, which also had specifically higher abundances (Figure 3B).

### 3.5 Discriminative Genera in SCC

To enhance the reliability of identifying and screening for genera that exhibit significant differential representation between tumor and adjacent tissue samples in SCCs, we employed the MaAsLin 2 method. The genera with significant differences are shown in Table S1.

**Figure 4:**
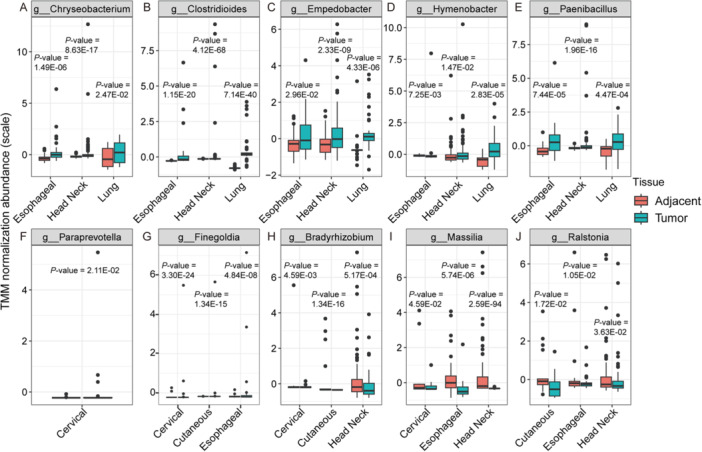
Boxplot illustrating the key bacterial genera with significant differences between tumor and adjacent tissue samples, with *p*‐values determined by the MaAsLin2 method. (A) *g__Chryseobacterium*, (B) *g__Clostridioides*, (C) *g__Empedobacter*, (D) *g__Hymenobacter*, (E) *g__Paenibacillus*, (F) *g__Paraprevotella*, (G) *g__Finegoldia*, (H) *g__Bradyrhizobium*, (I) *g__Massilia*, and (J) *g__Ralstonia*.

Although we did not identify the same significantly different genera across all five cohorts, we found some common genera in at most three SCCs, with ESCC, HNSCC, and LSCC shared the most up‐regulated genera, including *Chryseobacterium*, *Clostridioides*, *Empedobacter*, *Hymenobacter*, *Paenibacillus* and unclassified genera (Figure 4A–E, S2A). This implies that neighboring anatomical sites may harbor similar microbial characteristics, suggesting a commonality in the microbial signatures of these cancer types. For CeSCC and CuSCC, *Paraprevotella* and *Finegoldia* (also significantly upregulated in CeSCC and ESCC) were the most different up‐regulated genera in the two SCCs, respectively (Figure 4F, G). Among the significantly down‐regulated genera, only *Bradyrhizobium* (CeSCC, CuSCC, HNSCC), *Massilia* (CeSCC, ESCC, HNSCC), and *Ralstonia* (CuSCC, ESCC, HNSCC) were found in multiple cohorts (Figure 4H–J, S2B). These findings underscore the complexity and diversity of microbial communities associated with SCC from different sites.

### 3.6 Validation

**Figure 5:**
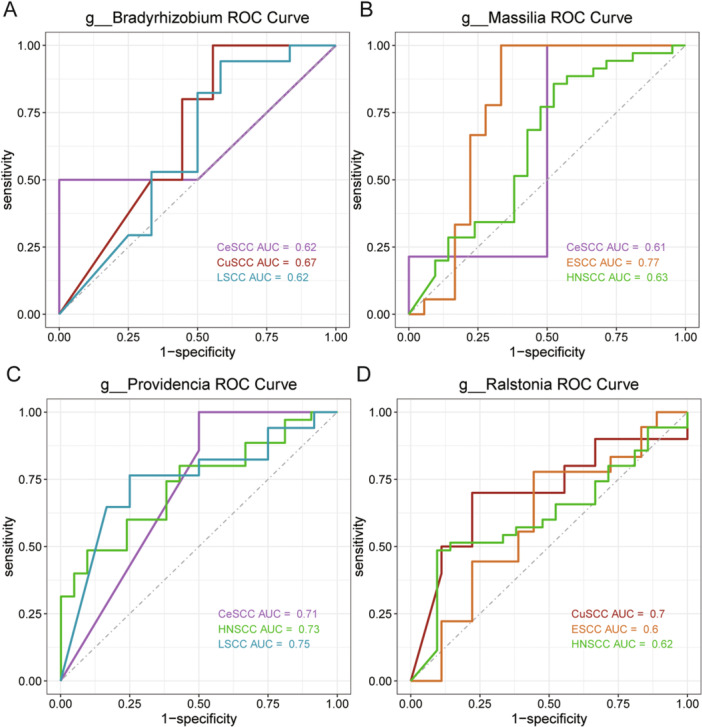
The ROC curves of significantly different genera. (A) *g__Bradyrhizobium*, (B) *g__Massilia*, (C) *g__Providencia*, and (D) *g__Ralstonia*.

To increase the reliability of the results, an additional dataset was selected in each cohort to validate the ability of discriminative genera to discriminate between tumor and adjacent normal tissue samples, and AUROC greater than 0.6 was considered to be a certain level of discriminatory ability for the genus. A total of 96 different genera confirmed the variability between tumor and adjacent tissue samples (Table S2). In particular, some genera common to multiple cohorts, such as *Bradyrhizobium*, *Massilia*, *Providencia*, and *Ralstonia* (Figure 5A–D).

## 4 Discussion

The advent of NGS technologies is providing new insights into understanding the human microbiome and its intricate role in cancer development. Genomics‐based studies have revealed that most major human cancers harbor an intratumoral microbiota [12]. These microbial communities differ across cancer types, and specific bacterial taxa may contribute to tumor initiation and progression, influence patients' treatment responses, and ultimately affect survival outcomes [13, 14]. For example, *Staphylococcus aureus*, induced dysbiosis has been associated with the development of CuSCC [15]; *Fusobacterium nucleatum* promotes immune evasion and PD‐L1 expression in ESCC [16]; enrichment of *Streptococcus* is a characteristic feature of LSCC [17]; the microbial features associated with immune types could predict the prognosis of HNSCC patients [18]; and in CeSCC, the predominance of *Bacteroides* and *Prevotella* reflects HPV‐associated microbial shifts [19]. It has been revealed that a similar mutational landscape among SCCs from different sites in the research on the cells of origin of SCCs [20]. However, it remains unclear whether SCCs arising from different anatomical sites share common or distinct microbial characteristics.

In this study, we used RNA‐seq data to dissect the microbiota within five distinct SCC cohorts. In CeSCC, no significant differences in microbial composition were observed between tumor and adjacent tissues, which differs from other types of squamous cell carcinoma. This phenomenon may be attributed to the fact that cervical carcinogenesis is primarily driven by persistent infection with high‐risk human papillomavirus (HPV), rather than by microbial dysbiosis. The cervicovaginal microbiota is generally dominated by *Lactobacillus* species and maintains a relatively stable ecological balance even during neoplastic transformation [21].

The further comprehensive analysis identified key bacterial genera, encompassing both core microbiota and differentially abundant taxa. We have identified both similarities and heterogeneities of microbiota among SCCs from different anatomical sites, which could potentially guide the development of both standardized and personalized treatment strategies for SCCs. Our findings not only corroborate but also expand upon previous research. For instance, *Staphylococcus*, the most prevalent and abundant bacteria in the SCC tissue microenvironment, has been implicated in our study. Notably, *Staphylococcus aureus* and its biofilm‐derived components, particularly alpha‐hemolysin (Hla), have been demonstrated to be selectively toxic to SCC cells [22]. This finding suggests a potential link between Staphylococcus abundance and targeted SCC therapies.

Recent studies have confirmed the core bacteria genera to be associated with PD‐L1 expression across various tumor tissues. For example, *Clostridium* [23], *Streptomyces* [24], and *Cutibacterium* [25] exhibit positive correlations with the expression of PD‐L1, while *Massilia* [26] is enriched in PD‐L1 negative samples, indicating the potential of these bacteria in either promoting or suppressing cancer within the tumor microenvironment. Although no common differential genera were identified across all five sites, the highest number of overlaps were found in the HNSCC, LSCC, and ESCC. This suggests that while the microbiota of SCCs from different anatomical sites exhibits some differences, there may be interactions between microbiota from neighboring sites, resulting in certain similarities. *Clostridioides* emerged as the most significantly different genera at the intersection of HNSCC, LSCC, and ESCC. *Clostridioides difficile*, a human pathogen widespread in the gastrointestinal tract and transmitted via the fecal‐oral route, supports our findings [27]. Studies have shown that *C. difficile* may induce colorectal cancer through chronic toxin‐mediated cellular alterations [28]. The remarkable clinical efficacy of fecal microbiota transplantation (FMT), proposed for *C. difficile* infection by current researchers, has also been observed in various cancer types [29], hinting at its potential role in the treatment of HNSCC, LSCC, and ESCC. *Providencia*, a new finding in this study, has not yet established a relationship with cancer development. In vivo studies have shown that *Providencia* can be transferred from the intestinal lumen to extra‐intestinal organs, confirming its potential role in the spread of infection [30]. Previous studies have also shown that an increase of *Providencia* could lead to the massive secretion of lethal swelling toxin, causing cell swelling, and blocking eukaryotic cell proliferation in the G2/M phase, resulting in cell death [31]. The significant enrichment of *Providencia* in multiple SCC tumor tissues in this study provides evidence for its association with cancer. Another bacterium of interest is the aerobic pathogen *Ralstonia*, which was significantly downregulated in cancer tissue samples from CuSCC, ESCC, and HNSCC. Lee et al. found that the abundance of *Ralstonia* increased following cosmetic use, suggesting that *Ralstonia* on facial skin may be capable of metabolizing cosmetic ingredients [32]. Certain strains of *Ralstonia* can break down aromatic hydrocarbons, trichloroethylene, and quinoline in contaminated environments, and the toxicity of the resulting degradation products is lower than that of the original compounds [33]. However, *Ralstonia* may influence the occurrence and development of tumors by participating in metabolic pathways such as the degradation of xenobiotics, which requires further investigation.

Our findings underscore the importance of understanding the specific microbiota associated with the occurrence and progression of SCC. The differential genera identified in our study may play a crucial role in the development and progression of SCC, warranting further research to explore their potential as diagnostic markers or therapeutic targets.

However, this study has a few limitations. First is the limited sample size. Therefore, the study integrated and analyzed both datasets to remove the batch effect to reduce the effect of individual heterogeneity on the results. Second is the potential for contamination in this type of study. Our study mainly focused on the analysis of core microbiota and verified the key bacterial genera in additional independent datasets to minimize the impact of potential contaminants on the study. Third, the cross‐sectional nature of this study limits our ability to establish temporal relationships or causality between microbial dysbiosis and SCC development. Fourth, while RNA‐seq allows simultaneous analysis of host transcriptome and microbiome, dedicated sequencing approaches (16S rRNA or shotgun metagenomics) may provide greater resolution of microbial communities. Future studies incorporating in vitro and in vivo functional experiments are warranted to further validate the roles of the identified key bacterial genera in SCC pathogenesis. Moreover, longitudinal analyses of microbiome dynamics during SCC initiation and progression may help clarify potential causal relationships. Finally, the potential biases that may be introduced by differences in gender or region should be further considered.

## 5 Conclusion

This study pioneers the exploration of the microbial compositional landscape within SCC tissues across five distinct anatomical sites: skin, esophagus, lung, head and neck, and cervix. Our findings reveal that *Staphylococcus* is a pervasive and abundant bacterium within the microenvironment of SCC tissues, warranting heightened attention due to its potential implications. The dysbiosis within the microenvironment of SCC tissues is influenced by the specific anatomical site. However, the greatest microbial similarity is observed among neighboring sites, namely esophagus, head and neck, and lung. Our results identify several bacteria (*Clostridioides*, *Providencia*, *Massilia*, and *Ralstonia*), that exhibit significant differences and may be involved in tumor progression, potentially serving as signature genera for SCC. These findings not only contribute to the understanding of the microbial landscape in SCC but also provide valuable data to support clinical translational applications. The distinct genera identified in this study could aid in early tumor screening and diagnosis, offering a foundation for further research. Moreover, these genera present promising candidate targets for experimental validation of the molecular mechanisms of microbial roles in SCC, highlighting the potential for microbiome‐based therapeutics and diagnostics in the future management of SCC.

## Author Contributions

All authors contributed to the study conception and design. Material preparation, data collection, and analysis were mainly performed by Ruiqian Yao. Liangzhe Wang and Yuanjie Zhu were major contributors to the design of this study and revised the manuscript. The first draft of the manuscript was written by Ruiqian Yao, Fang Qian and all authors commented on previous versions of the manuscript. All authors read and approved the final manuscript.

## Ethics Statement

The authors have nothing to report.

## Consent

All authors have read and approved the content and agree to submit for consideration for publication in the journal.

## Conflicts of Interest

The authors declare no conflicts of interest.

## Supporting information


**Figure S1:** PCoA plots clustered by project ID, based on Bray‐Curtis dissimilarity on raw count data (left) and ConQuR corrected data (right).

**Figure S2:** The overlap of up (A) and down‐regulated (B) bacteria in the five cohorts.

**Table S1:** Significantly different genera.

**Table S2:** Significantly different genera with AUROC > 0.6 in the validation cohort. Red represents equally significant differences in other cohorts.

## Data Availability

The dataset analyzed in this study is available at NCBI (http://www.ncbi.nlm.nih.gov/).
