# Supplementary figures and images for: Microbiome Landscapes in Squamous Cell Carcinoma Tissue Microenvironments: A Comparative Analysis
Source: Immun Inflamm Dis. 2026 Mar 17;14(3):e70406. doi: 10.1002/iid3.70406 (PMC13097533; doi:10.1002/iid3.70406)

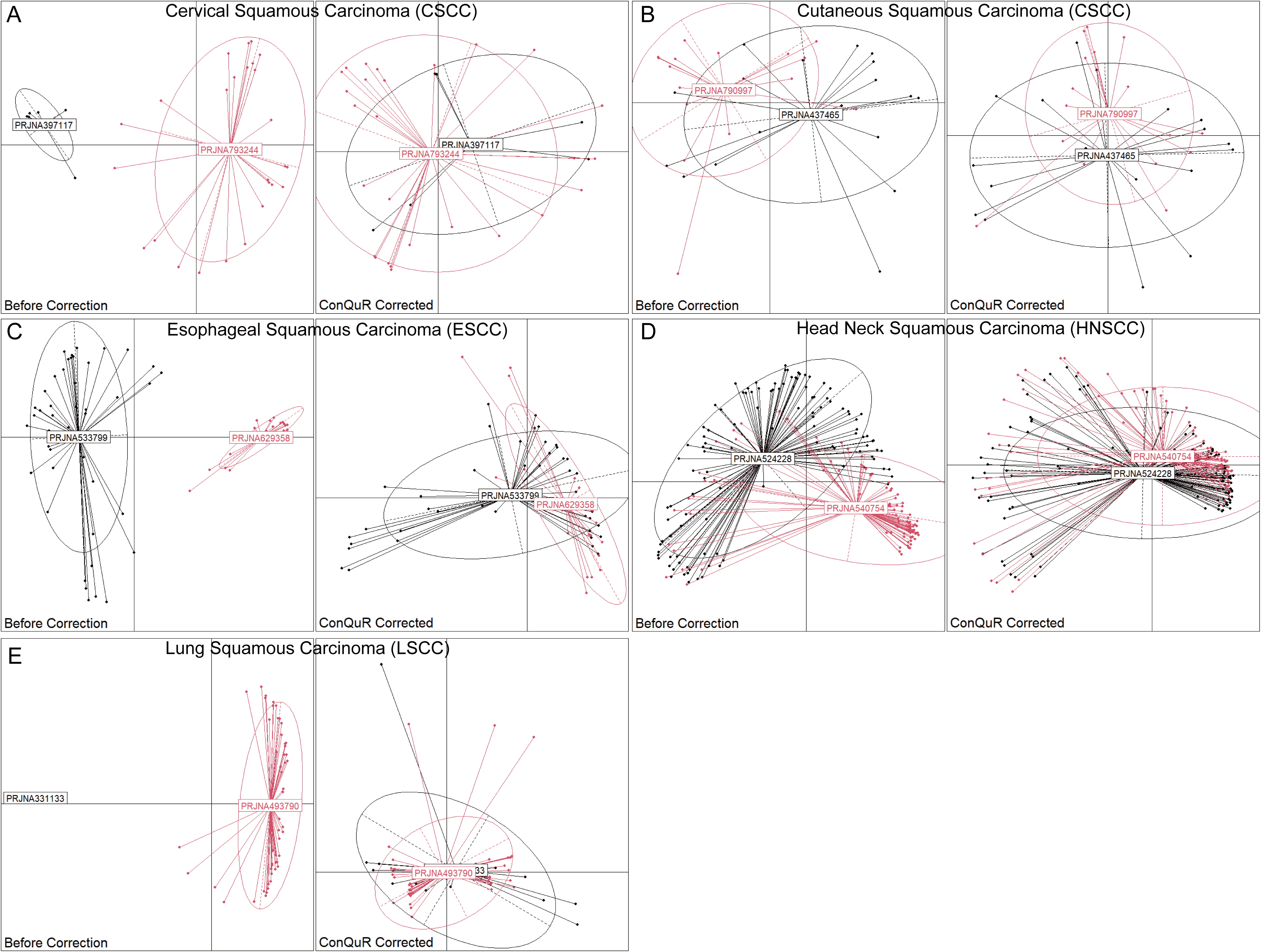

Supplement: Supplementary file 1 — Figure S1: PCoA plots clustered by project ID, based on Bray‐Curtis dissimilarity on raw count data (left) and ConQuR corrected data (right). [file IID3-14-e70406-s002.tif]

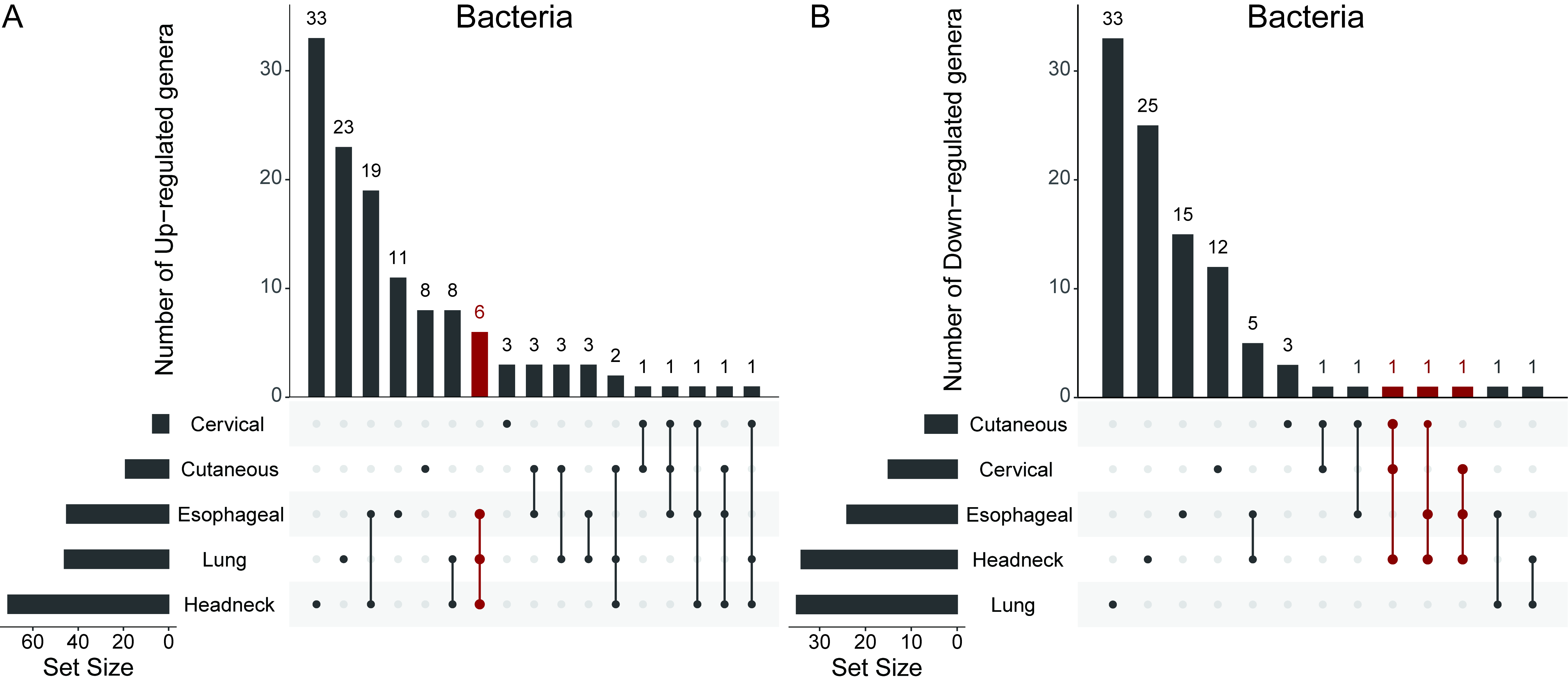

Supplement: Supplementary file 2 — Figure S2: The overlap of up (A) and down‐regulated (B) bacteria in the five cohorts. [file IID3-14-e70406-s001.tif]
